# Laser-Induced Biochar Formation through 355 nm Pulsed Laser Irradiation of Wood, and Application to Eco-Friendly pH Sensors

**DOI:** 10.3390/nano10101904

**Published:** 2020-09-24

**Authors:** Sung-Yeob Jeong, Chan-Woo Lee, Jun-Uk Lee, Yong-Won Ma, Bo-Sung Shin

**Affiliations:** 1 Interdisciplinary Department for Advanced Innovative Manufacturing Engineering, Pusan National University, Pusan 46241, Korea; ysjsykj8025@naver.com (S.-Y.J.); decentsoul@pusan.ac.kr (Y.-W.M.); 2 Department of Cogno-Mechatronics Engineering, Pusan National University, Pusan 46241, Korea; cwleeho2@naver.com (C.-W.L.); lju3534@naver.com (J.-U.L.); 3 Department of Optics and Mechatronics Engineering, Pusan National University, Pusan 46241, Korea

**Keywords:** biochar, 355 nm pulsed laser, lignin, pH sensor, eco-friendly

## Abstract

Due to the limited availability of agricultural land, pH sensing is becoming more and more important these days to produce efficient agricultural products. Therefore, to fabricate eco-friendly and disposable sensors, the black carbon, which is called biochar, is formed by irradiation of a UV pulsed laser having a wavelength of 355 nm onto wood and applying the resulting material as a pH sensor. The surfaces of three types of wood (beech, cork oak, and ash) were converted to the graphitic structure after UV laser irradiation; their morphologies were investigated. In addition, since the content of lignin, an organic polymer, is different for each wood, optimal laser irradiation conditions (laser fluence) needed to form these woods into pH sensors were considered. Depending on the degree of oil-like material generated after laser irradiation, a disposable pH sensor that can be used from one to three times is fabricated; due to the environmental characteristics of wood and biochar, the sensor shows high availability in that it can be easily discarded after use on agricultural land. After that, it can be used as filter in soil. Our wood-based pH sensor sensitively measures sequential changes from pH 4 to pH 10 and shows a very linear change of △R/R, indicating its potential for use in agriculture.

## 1. Introduction

Recently, studies on fabricating graphitic structure by irradiating a laser under specific conditions on commercial polyimide films (PI, Kapton^®^) have been actively published [1,2,3]. Many papers have been published delineating the use of the graphitic structure, fabricated by irradiating a laser on polyimide, as platforms for supercapacitors, physical sensors, and humidity sensors [4,5,6,7]. We previously reported a strain sensor in which electrical changes occur with strain induced by irradiating polyimide (Kapton^®^, HN) with a 355 nm ultraviolet pulsed laser. Furthermore, in recent years, graphitic materials derived from organic polymers, such as cloth, paper, potato, coconut, cork, and wood have been actively reported [2,8]. Compared to previously discovered polymer precursors, these organic polymers are renewable, inexpensive, easily found around us, biodegradable, and eco-friendly. Above all, it is important to form a flat surface so as to minimize laser focus changes on the non-uniform surfaces [2]. In order to irradiate the laser onto a flat sample surface, among various organic polymers, we conducted an experiment using wood. A renewable resource, wood is used even today as an indoor building material and furniture material due to its aesthetic patterns, ease of processing in various forms, and excellent mechanical properties. Moreover, wood is an excellent source for the development of electrochemical devices and microelectromechanical systems [9,10]. In this paper, we report the fabrication of an graphitic pattern, which is called biochar, with high electrical properties by scribing a 355 nm ultraviolet pulsed laser onto three different types of wood (beech, cork oak, and ash) [11]. The biochar generated from the pyrolysis of biomass not only improves soil quality and reduces greenhouse gas emissions from the soil but also reduces the toxicity of metals in the soil and helps the more efficient use of phosphorus and potassium [12,13,14]. Here, we report on a sensor made with biochar fabricated on a wood surface for application to the smart farm industry. As available farmland cannot be increased, harvesting crops effectively within a limited area is the biggest issue in the field of agriculture [15]. Nitrogen (N), phosphorus (P), and potassium (K) are the main macronutrients, the three most important nutrients for plants [15,16,17,18]. Inadequate content of nitrogen (N), phosphorus (P), and potassium (K) reduces crop quality and quantity. Nitrogen is important in the amino acids that are the basis of proteins [19,20,21]. Phosphorus is an important component of the complex nucleic acid structure of plants and plays an important role in cell division and the formation of new tissues [21]. Phosphorus is associated with plant tolerance, root growth, and complex energy exchange. Potassium is the most important nutrient for crop quality [19,20,21]. High content of these three nutrients enhances plant defenses and improves the shape, color, taste, and shelf life of fruits and vegetables [14,20]. Another pivotal point is that agriculture is the pH concentration of the nutrient soil [21]. When the soil becomes acidic, iron (Fe) and aluminum (Al) combine with phosphoric acid and the resulting iron phosphate and aluminum phosphate cannot be absorbed by plants; nitrogen become nitrous acid and evaporates into the air. By monitoring the pH level of the soil, crop productivity can be improved. Consequently, to measure pH level, we used an biochar platform to fabricate a disposable organic eco-friendly sensor. More specifically, we report an eco-friendly organic polymer-based pH sensor and suggest its suitability for use in smart farms.

## 2. Materials and Methods

### 2.1. UV Pulsed 355 nm Laser System

**Figure 1:**
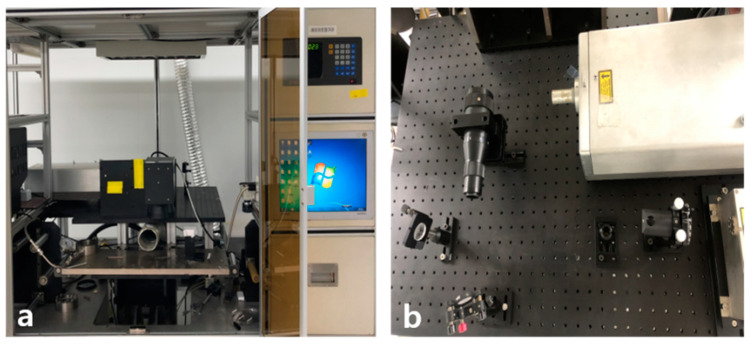
(**a**) Laser set-up; (**b**) optical system.

In this study, a graphitic pattern was fabricated by irradiation of a 355 nm UV pulsed laser (AONano 355-5-30-V from Advanced Optowave, Ronkonkoma, NA, USA). The 355 nm UV pulsed laser system we used is shown in Figure 1. Among lasers of UV wavelength, the 355 nm nanosecond laser has very short wavelength characteristics and high power [22,23]. UV laser micromachining is a very attractive process for biodegradable polymeric materials because the laser can minimize thermal effects transmitted to the processed material as the wavelength of the beam becomes shorter, greatly reducing the thermal damage to unprocessed areas [22,24,25]. As a result, such lasers are widely used for industrial cutting and surface modification, offering stable operation and high productivity and utilization of work surfaces.

The 355 nm UV pulsed laser is a high-powered laser of ultraviolet wavelength; it has the advantage of stability when used for micro-processing of polymers and is widely used in many industry fields. The laser specifications are shown in Table 1.

### 2.2. Lignin in Wood

**Figure 2:**
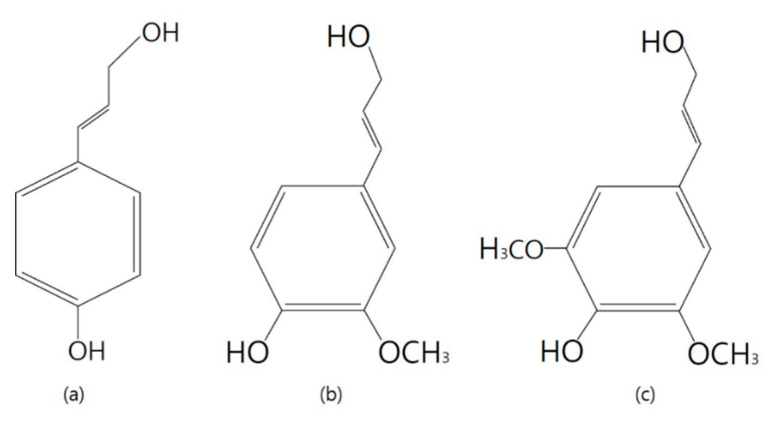
Chemical structure of lignin precursor; (**a**) p-coumaryl alcohol (H), (**b**) coniferyl alcohol (G), and (**c**) sinapyl alcohol.

As shown in the Figure 2, wood has an organic polymer called lignin, which forms an important structural material that supports tissues in certain algae and vascular plants. Lignin is a precursor of p-coumaryl alcohol (H), coniferyl alcohol (G), and sinapyl alcohol (s), forming a complex three-dimensional polymer by β-O-4 or carbon–carbon bonding [26,27,28].

**Figure 3:**
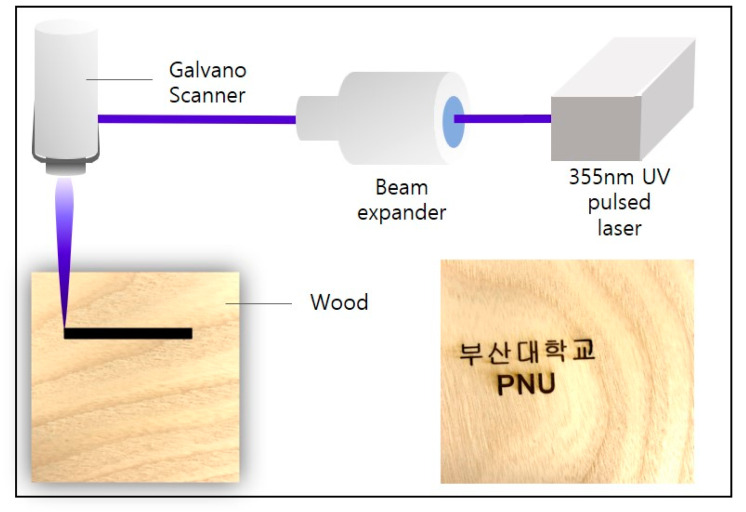
Schematic diagram of laser irradiated on surface of wood.

Under certain conditions, these lignin-containing precursors can be converted to graphitic structure by laser irradiation [2,12]. In addition, because the conversion to biochar is determined by the lignin content of the wood, lignin, which is a heterogeneous aromatic polymer, is a decisive factor in the manufacturing of biochar by irradiating wood with a laser [12]. We utilized a direct laser writing (DLW) method to irradiate a 355 nm ultraviolet pulsed laser on wood samples with different lignin contents, including ash, with low lignin content of 7.2%, cork oak (23.6%), and beech (25.5%). A direct laser writing (DLW) method using a Galvano scanner (hurrySCAN III 14, SCANLAB, Pucheim, Germany) can quickly produce biochar in a single step. Figure 3 depicts the scheme for the fabrication of biochar on wood. The resulting graphitic pattern on the wood surface can be easily and simply patterned into various shapes by computer design. According to previous structural studies, the higher the lignin content is in the wood containing lignocellulose, the better the synthesis of high-quality graphite or graphitic layers [2,12,29].

### 2.3. Fabricaton of Biochar

Figure 3 shows that the surface has been successfully converted to biochar even though laser irradiation was performed under ambient conditions without the use of an inert gases, such as argon. This biochar fabricating process simply overcomes the disadvantages of the conventional graphitic layer manufacturing process and the demerits of wood-based graphitic material, which had to be irradiated with a laser under inert gas conditions [12]. The fabricated biochar on the wood surface was patterned by computer design and produced in the desired shape using a Galvano scanner. Each of the wood samples used in the experiment was found to form a graphitic pattern with optimal electrical properties, such as low sheet resistance, high capacity, and good I-V characteristics, when laser irradiation was performed at specific laser fluences of 20.37 mJ/cm^2^ (beech), 14.12 mJ/cm^2^ (cork oak), and 23.14 mJ/cm^2^ (ash). The dimension of the carbonized area is 4 × 10 mm, which was irradiated with a scan speed of 40 mm/s. Certain threshold laser fluence was required to make each wood convert biochar (beech wood: 9.28 mJ/cm^2^, cork oak wood: 4.34 mJ/cm^2^, and ash wood: 10.99 mJ/cm^2^). In addition, from these threshold laser fluence, the higher the pulse energy density, the higher the degree of carbonization.

### 2.4. Appying Organic Polymer-Based Sensor to Agriculture

**Figure 4:**
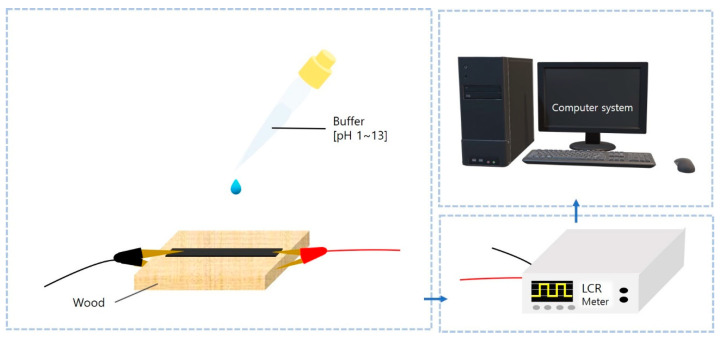
Schematic diagram of pH buffer being carefully dropped onto natural polymer-based sensor.

Our organic polymer-based sensor was fabricated simply by using silver paste to connect electrodes for pH sensing and electrolyte concentration sensing. Unlike the annealing process and the complex manufacturing process that conventional graphitic material-based sensors have to undergo, our sensor, which has a quick and simple fabricating process, has been used to conduct various types of sensing tests according to the type of wood. Depending on the amount of oil-like material produced by irradiating the UV laser on the wood, the numbers of iterations of pH sensing and electrolyte sensing were set as follows: beech: 2 times/cork oak: 3 times/ash: 1 time). These oil-like substances called wood tar extracted from the formed due to wood pyrolysis. As can be seen in Figure 4, the pH buffer was carefully dropped dropwise onto the sensor and had a stabilization time of about 1 min. After that, the pattern functions as a pH sensor based on the chemiresistor principle. Furthermore, the change of electrical properties of our sensor was measured with an LCR meter (LCR meter 4410, Keithley Tektronics, Beaverton, OR, USA).

## 3. Results and Discussion

### 3.1. Graphitic Pattern Formed on Wood

Here, our biochar, fabricated using simply the photo-thermal and -chemical effects of wood generated by irradiation of a 355 nm ultraviolet pulsed laser, easily overcomes the demerits of high cost and high manufacturing time necessary in conventional graphene manufacturing. Figure 3 demonstrates that our graphitic logo pattern can be plotted by a computer program and freely scanned on the wood samples through a Galvano scanner.

#### 3.1.1. Morphology

The morphology of the graphitic pattern fabricated simply by laser irradiation of the wood samples under ambient conditions without inert atmosphere has been observed by scanning electron microscopy (SEM, TESCAN (VEGA II LSU), Brno, Czech Republic). In addition, SEM images of pristine woods are shown in Figure S1.

**Figure 5:**
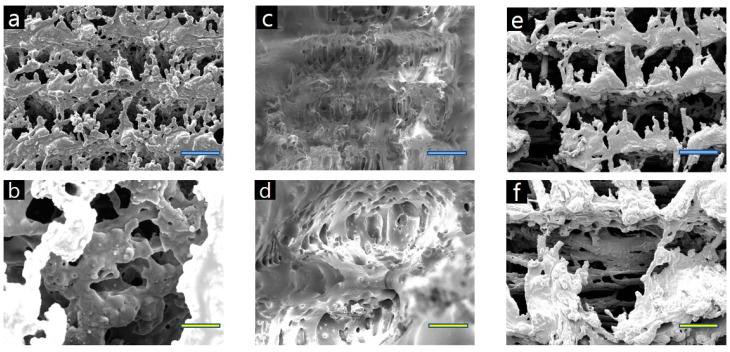
SEM images of wood surfaces carbonized by laser irradiation: (**a**,**b**) beech, (**c**,**d**) cork oak, (**e**,**f**) ash; blue scale bar: 100 μm; yellow scale bar: 20 μm.

**Figure 6:**
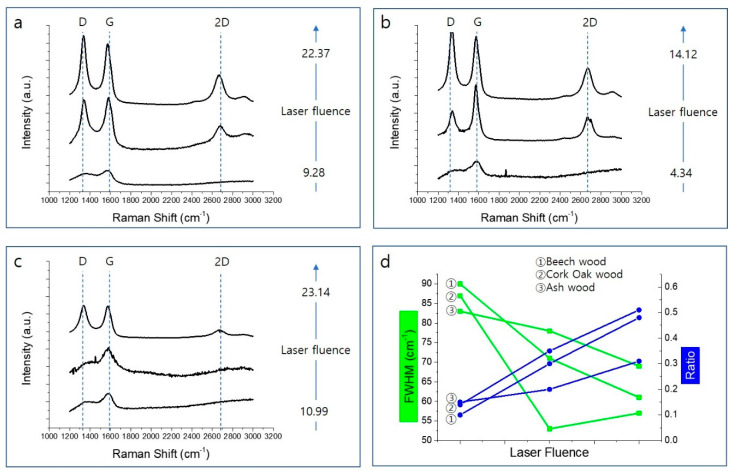
Changes in characteristics of beech, cork oak, and ash wood according to laser fluence; (**a**–**c**) Raman spectrum of three wood surfaces, (**d**) full-width-at-half-maximum (FWHM) and I2D/IG ratio of G peak according to laser intensity.

Figure 5a–f shows how the surfaces of beech, cork oak, and ash wood change with the given values of laser fluence. A quantity of oil-like material is shown to be generated in descending order of cork oak, beech, and ash wood; this oil clearly controls the amount of moisture absorbed by the wood and strongly influences the test of dropping liquid onto the surface. Figure 5 shows that the surface of each wood sample has a porous structure after laser irradiation at a specific laser fluence; each laser fluence level was applied to the wood samples through a change in scanning speed. Until the laser is applied at the optimal laser fluence, as the laser fluence increases, the surface of the wood becomes more and more porous and hierarchical. Moreover, laser irradiation induces material carbonization making the substrate conductive. The content of conductive carbon sp^2^ species could be explored by analyzing C KLL Auger spectra or analyzing Raman spectrum [30,31]. As shown in Figure 6, chemical structural analysis of our organic polymer-based sensor was investigated through Raman spectroscopy analysis. Until the optimal laser fluence value to convert biochar on the surface of each wood sample was more and more distinct, Raman signals were observed as the laser fluence increased (Figure 6a–c). The D peak at ~1350 cm^−1^ is associated with a disorder in sp^2^ hybridized core. In our materials, it has great intensity because the structure has more defects/replacement points. The G peak at ~1580 cm^−1^ is produced by stretching in sp^2^ system and, therefore, is essential for graphite-related materials. The 2D peak (~2700 cm^−1^), which is called the overtone mode of the D mode, is known to enhance the signal due to the double resonance phenomenon, making it is easy to confirm the layer thickness in the graphene and graphite crystals. In addition, in general, a material in which n-layers of graphene are laminated has a peak intensity proportional to the intensity thickness of the G peak, and the line shape of the 2D peak also changes according to the thickness.

For the analysis of the graphene structure, the full-width-at-half-maximum (FWHM) of the G peak and the I_2D_/I_G_ ratio were investigated. As the laser fluence increases, the I_2D_/I_G_ ratio increases and the FWHM of the G peak increases, which means that stacking fewer graphitic layers and maximized crystalline size of graphitic materials. This is attributed to the fact that this phenomenon results from the higher quality graphene-like material produced. In addition, the more lignin-rich wood (in the order of beech, cork oak, ash), the more pronounced the peaks are in the Raman spectrum. These results indicate that the surface of the wood can be successfully converted to biochar with a 355 nm UV pulsed laser, even without specific inert gas conditions. The comparison of the molecular structure of the biochars generated and each wood before laser irradiation was analyzed by Fourier transform infrared spectrometer (FT-4100 JASCO, Easton, MD, USA). As shown in Figure S2, the peak at 3413 cm^−1^ is characterized by the –OH hydrogen bond in wood, the peak at −2927 cm^−1^ is assigned to the CH_3_ (Methyl) and CH_2_-(methylene) groups in the wood, and 1217 cm^−1^ is assigned to interrogator ring breathing using CO and C–O stretching [32]. Notable change here is peak at 2344 cm^−1^, which indicates formation of CO_2_ [33] and more graphitic structure is formed by 355 nm pulsed laser irradiation.

**Figure 7:**
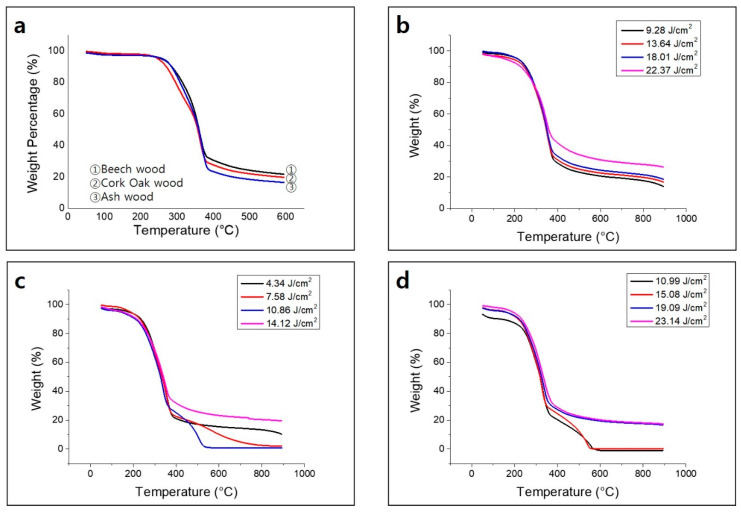
Thermogravimetric analysis (TGA) of (**a**) beech, cork oak, and ash wood and biochar derived from (**b**) beech, (**c**) cork oak, and (**d**) ash wood.

**Figure 8:**
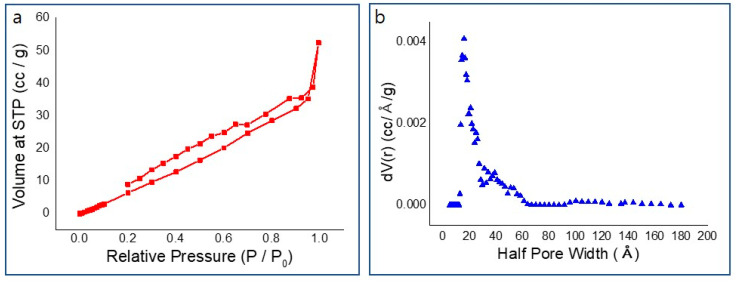
(**a**) N_2_ adsorption/desorption isotherms; (**b**) Barrett–Joyner–Halenda pore size distribution plot of beech wood.

Thermogravimetric analysis (TGA) results suggest that the weight loss at around 100 °C is caused by evaporation of moisture on the surface and inside the wood (Figure 7a). The large weight loss in the temperature range of 240–400 °C results from the decomposition of aliphatic and aromatic carbon at ~240 °C and ~320 °C, respectively [34]. Lignin is composed of a high-molecular-weight substance mainly containing an aromatic polymer substance composed of strongly crosslinked constituent units. Therefore, it requires a relatively high thermal decomposition temperature compared to cellulose and hemicellulose. Figure 7a shows that beech wood (25.5%) with high lignin content, has higher thermal stability than cork oak wood (23.6%) and ash wood (7.2%). Figure 7b–d shows the change in thermal stability according to the laser fluence. As can be seen in Figure 7b–d, thermogravimetric analysis (TGA) results in air suggest that increasing the laser fluence enhances the thermal stability of beech, cork oak, and ash wood. The BET surface area of beech wood was measured by N_2_ adsorption and desorption technique based on the Barrett–Joyner–Halenda model (Figure 8a). Figure 8b shows the calculated pore size distributions. N_2_ adsorption-based surface area analysis yielded a value of 132.968 m^2^/g of BET surface area; average pore radius is 12.18 Å.

#### 3.1.2. pH Sensor

**Figure 9:**
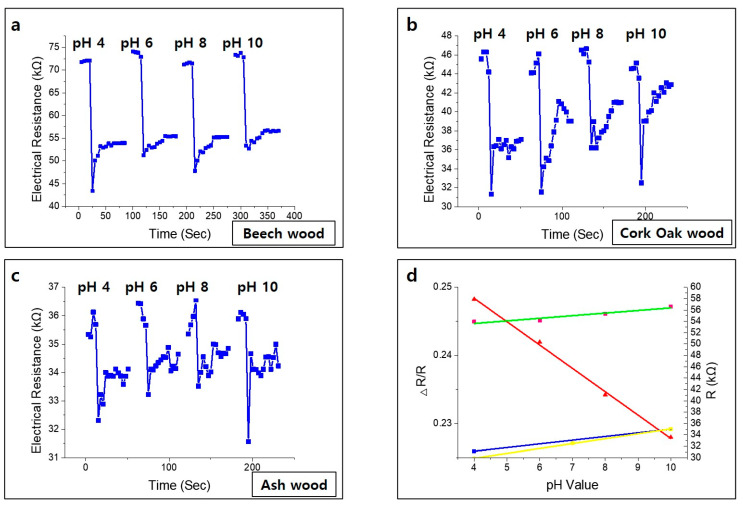
Change of sensor electrical conductivity according to pH value with a single buffer drop test; (**a**) beech wood, (**b**) cork oak wood, (**c**) ash wood, (**d**) investigation of linearity of changes in electrical properties of beech wood-based and changes in electrical resistance of each sensor according to pH value.

pH measurement is necessary for bio-sensing, environmental monitoring, clinical diagnosis, water quality measurement, and soil measurement [35,36,37,38,39,40,41]. Nan Lei et al. reported the fabrication and characterization of a simple gate-free graphene device as a pH sensor. It has been shown that the resistance of the device decreases linearly (in the pH range of 4–10) as the pH value increases in the surrounding liquid environment [42]. Rinky Sha et al. demonstrated a Gr-PANi composite-based amperometry pH sensor. The as-fabricated pH sensor exhibits short response time and excellent sensitivity of −50.14 μA pH^−1^ cm^−2^ in the range of pH 1–5 and 139.2 μA pH^−1^ cm^−2^ in the range of pH 7–11 [43]. These conventional pH sensors include a post-treatment annealing process that is complex, time-consuming, and expensive. We report very sensitive, eco-friendly, disposable, and low-cost manufacturable sensors that show different electrical resistance levels depending on the pH value. Figure 5 shows that the surface of each wood sample has a porous structure after laser irradiation at a specific laser fluence; each laser fluence was applied to the wood by changing the scanning speed. The analysis system was connected to the sensor, and the change in electrical resistance was measured in real time by an LCR meter. After using a nanopipette to carefully drop the pH buffer into the organic graphitic pattern, and after stabilization time of about 1 min, various concentration pH measurement tests were performed, with results shown in Figure 9.

When irradiated with a laser, beech and ash wood have relatively less oil-like materials than does cork oak wood, and the pore size of their graphitic patterns is large; so, when the pH buffer was dropped, sensors made from beech and ash wood did not recover to their original states after 1–2 experiments. As shown in Figure 9a, the wood-based sensors formed on the surface of the beech wood exhibited a linear change in electrical properties in the range of pH 4 to 10. Likewise, other wood-based pH sensors (cork oak wood and ash wood) can detect the change of the pH value sensitively as well (Figure 9b–c). A linear relationship is observed between the standard resistance and the pH value from 4 to 10. As shown in Figure 9d, when the pH value increases, the resistance of the sensor increases. The normalized resistance, defined as Equation (1)

(1) $$\frac{\Delta R}{R}=\frac{R-R_{min}}{R_{max}-R_{min}}$$

is used to evaluate the performance of wood-based sensors, where Δ*R*, *R*, *R_max_* and *R_min_* are the real-time sensor resistance relative to its lowest value, the range of resistance in the entire measurement scheme, the highest resistance, and the lowest resistance, respectively. Real-time resistance changes at different pH values demonstrate that the sensor is highly sensitive to pH changes. All curves show an immediate reaction after the buffer solution covers the wood-based sensor.

**Figure 10:**
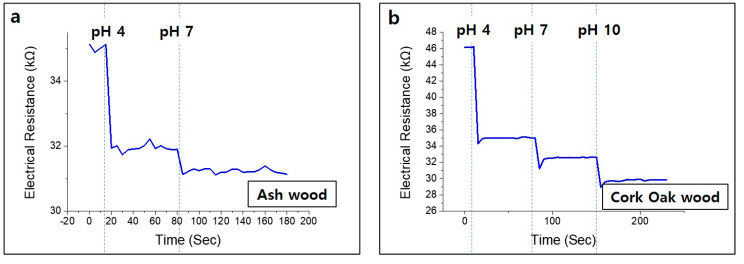
Change of sensor electrical conductivity according to pH value with a multiple buffer drop test; (**a**) ash wood (two times), (**b**) cork oak wood (three times).

Figure 10b shows the three different pH buffer drops on cork oak wood. The first pH 4 buffer drop was carefully placed on top of cork oak wood sample. Then, after a stabilization time of 1 min, pH 7 and pH 10 buffers were sequentially dropped onto the sample. A decrement of the electrical resistance was observed when the buffer was changed to pH 4, pH 7, and pH 10. The sensor made of cork oak wood was valid until the third experiment when the pH buffer was dropped. Ash wood produced relatively smaller amounts of oil-like substances, which is called wood tar, than did beech or cork oak, so it did not recover to its original state after one experiment (Figure 10a). When a drop of pH 7 buffer was placed on top of the ash wood, after dropping of the pH 4 buffer, the resistance rapidly decreased. Wood-based sensors have superb responses to pH changes, providing high potential for real-time sensing applications. We report very sensitive, eco-friendly, disposable, low-cost process pH sensors that are simple to manufacture. The pH sensing principle can be explained by the adsorbed ions on the inner Helmholtz plane. The adsorption of H^+^ or OH^−^ at the inner Helmholtz plane is non-Faradaic, and so the charges cannot transmit across the graphene interface. Both hydroxyl ions (OH^−^) and hydroxonium (H_3_O^+^) ions are adsorbed on the graphene surface, according to the configuration of the electrical double layer of graphene. As the concentration of H^+^ ions is higher in the acidic region, H^+^ ions are adsorbed at the inner Helmholtz plane, which attracts electrons and becomes n-doped. Likewise, in the alkaline region, the concentration of OH^−^ ions is higher; H^+^ ions are adsorbed at the inner Helmholtz plane, which attracts holes and becomes p-doped.

## 4. Conclusions

This paper reports on a study of the characteristics of biochar on the surface of three kinds of wood converted by irradiation with a 355 nm UV pulsed laser according to the content of lignin, the organic polymer of wood. An application of this process for the formation of pH sensors is detailed. The main motivation for this experiment was that there is no efficient method of raising crops in limited farmland in the modern agricultural environment. The direct laser writing (DLW) technique using a 355 nm UV pulsed laser can produce biochar simple in a single step, enabling application of the material as a pH sensor. This process makes it possible to manufacture large numbers of sensors inexpensively and quickly. The graphitic pattern can be fabricated within very short time (beech wood- and cork oak wood-based sensor: ~280 s and ash wood-based sensor: ~70 s). The biggest advantage of this pH sensor is that it is wood-based, making it environmentally friendly and disposable. After that the sensor can be utilized as biochar in the soil so that it not only improves soil quality and reduces greenhouse gas emissions from the soil, but also reduces the toxicity of metals in the soil and helps the more efficient use of phosphorus and potassium. After laser irradiation, quantities of oil-like materials, which are called wood tar, were formed descending order of cork oak, beech, and ash wood; the larger the quantity of this material, the smaller the pore size, and the more difficult it is for moisture to penetrate. In addition, our sensor showed a very linear △R/R, which indicates the good sensitivity of the pH sensor, making it possible to measure the change of pH value in real time.

## Figures and Tables

**Table 1:** UV pulsed 355 nm laser specifications.

Parameter	Value	Unit
**Wavelength**	355	nm
**Average power**	~2.5	Watt
**Pulse length**	25	nsec
**Repetition rate**	30	kHz

## Supplementary Materials

The following are available online at https://www.mdpi.com/2079-4991/10/10/1904/s1, Figure S1: SEM images of pristine wood surfaces, Figure S2: Fourier transform infrared spectroscopy of three pristine woods and biochar from each wood, Video S1: pH sensing test.
